# Sustainable Materials from Organosolv Fibers and Lignin, Kraft Fibers, and Their Blends

**DOI:** 10.3390/polym16030377

**Published:** 2024-01-30

**Authors:** Jost Ruwoldt, Gary Chinga-Carrasco, Mihaela Tanase-Opedal

**Affiliations:** RISE PFI AS, Høgskoleringen 6B, 7491 Trondheim, Norway; gary.chinga.carrasco@rise-pfi.no (G.C.-C.); mihaela.tanase@rise-pfi.no (M.T.-O.)

**Keywords:** organosolv fibers, thermoforming, molded pulp, Kraft pulp, green materials, added-lignin thermoformed pulps

## Abstract

The aim of this study was to investigate new materials from organosolv fibers, organosolv lignin, kraft fibers, and their blends. The organosolv fibers showed reprecipitated lignin on the surface, a comparably low fiber length of 0.565 mm on average, and a high fines content of 82.3%. Handsheets were formed and thermopressed at 175 °C and 50 MPa, yielding dense materials (1050–1100 kg/m^3^) with properties different to that of regular paper products. The thermopressing of organosolv fibers alone produced materials with similar or better tensile strength (σ_b_ = 18.6 MPa) and stiffness (E* = 2.8 GPa) to the softwood Kraft reference pulp (σ_b_ = 14.8 MPa, E* = 1.8 GPa). The surface morphology was also smoother with fewer cavities. As a result, the thermopressed organosolv fibers exhibited higher hydrophobicity (contact angle > 95°) and had the lowest overall water uptake. Combinations of Kraft fibers with organosolv fibers or organosolv lignin showed reduced wetting and a higher density than the Kraft fibers alone. Furthermore, the addition of organosolv lignin to Kraft fibers greatly improved tensile stiffness and strength (σ_b_ = 23.8 MPa, E* = 10.5 GPa), likely due to the lignin acting as a binder to the fiber network. In conclusion, new thermopressed materials were developed and tested, which show promising potential for sustainable fiber materials with improved water resistance.

## 1. Introduction

Current environmental demands and the shift towards green technologies have motivated an extensive valorization of biomass. Here, great emphasis is put on the development of biobased, renewable, and biodegradable products to substitute fossil-based plastics and hence reduce microplastics pollution. The EU directive “Single-use plastic” has the main objectives of preventing and reducing the impact of plastics on the environment and promoting a more circular bioeconomy [1]. As such, finding sustainable alternatives has become one of the main concerns as of today. Biorefineries serve as a platform to convert lignocellulosic biomass to chemicals and fuels. In a circular bioeconomy, the maximized utilization of lignocellulosic biomass and the minimization of waste and emissions are very well integrated in a biorefinery concept [2]. 

Lignocellulosic fibers, e.g., chemical, chemi-thermomechanical (CTMP), and thermomechanical pulp (TMP), have been extensively explored for thermoformed materials [3,4,5,6]. However, two of the main challenges with lignocellulosic fibers remain, which are the hydrophilicity and lack of water resistance. These properties can limit the applicability of lignocellulosic fibers in products, where a liquid barrier is required, e.g., beverage and food packaging materials. Traditionally, paperboard packaging materials are coated with synthetic polymers that enhance their resistance to water, moisture, grease, oxygen, and odor [7]. On the other hand, it has been shown that biopolymers can potentially reduce carbon dioxide emissions by 30 to 70% [8], induce hydrophobicity, and promote inter-fiber adhesion and mechanical strength [9]. Lignin, the second-most abundant biopolymer from lignocellulosic biomass, is considered a byproduct from various biorefinery processes, which is still underutilized as of today. Hence, because of its abundance and chemical structure, lignin has a significant potential to be used in many applications such as biopolymeric materials [10], reinforced filler materials [11], bioactive components [10], and internal sizing agents [12]. As such, the application of lignin in thermoformed fiber materials is a promising alternative, which can improve the properties of fiber-based materials, while additionally creating added-value applications for lignin in fully biobased products. 

The organosolv process enables the efficient fractionation of lignocellulosic biomass, e.g., by removing lignin and hemicelluloses to produce cellulose fibers [13]. Moreover, organosolv pulping of lignocellulosic biomass can be designed to obtain cellulose fibers with reprecipitated lignin on fiber surfaces [14]. This process may seem unsuitable for conventional applications of cellulose fibers, such as paper, textile, and tissue products. However, in thermopressed materials, reprecipitated lignin on the fiber surfaces can be an advantage, as lignin could provide strength and binding ability, while reducing wetting. Purified organosolv is a sulfur-free lignin with a high degree of reactivity and purity, lower molecular weight, and lower polydispersity when compared to other technical lignins [15]. In more general terms, technical lignins have been reported to provide both gas and UV light barrier properties, as well as antimicrobial coatings [16,17,18]. 

Molded pulp products can be categorized into type 1 “thick walled”, type 2 “transfer molded”, and type 3 “thin walled” [19]. The latter is made through vacuum filtration of a pulp suspension into a shaped geometry, transfer into a heated mold, and subsequent pressing under high temperature and pressure. This process is also referred to as thermoforming, whereas in this study, thermopressing describes the same principle with the limitation to planar 2D substrates. The advantages of thermoforming include improved material stiffness and strength, as well as a better resistance to moisture and reduced wetting [3]. Lignocellulose fibers can hence be processed into materials that resemble the properties of fossil-based plastics more closely, while not contributing to microplastics pollution. In particular, the addition of technical lignin can promote such material improvements [4,12].

The mechanical strength of thermoformed materials reportedly stems from hydrogen bonding between the fibers, condensation reactions of lignin, and the partial degradation of hemicellulose [20]. It has been shown that approximately 6–17% residual lignin in the fibers may distinctly contribute to the strength, stiffness, and water resistance of molded pulp products [21]. Early attempts to increase the lignin content above natural levels of wood-pulp products include films, which were based on lignosulfonate–starch blends [22]. Here, lignosulfonate may contribute to the improved strength and water resistance of starch coatings, e.g., used for corrugated cardboard. Another invention from the same decade includes Arboform^®^ [23], which combined lignocellulose fibers with lignosulfonate and other additives to produce a thermoplastic material. Such pellets can be injection-molded into various shapes, hence deviating in technology from thermoforming, while providing a similar fiber–lignin matrix. Lignin has also been researched as an alternative binder in fiber boards, which may provide an economic alternative to traditional binders [24,25]. Besides employing a coarser fiber type than traditional pulp, fiber board production also utilizes heat and pressure to densify the material, and there are hence analogies to molded pulp materials. More recent studies have also incorporated traditional pulp fibers, such as TMP or chemical pulp [26,27].

The addition of lignin to paper products has predominantly been carried out by surface-sizing. A systematic study into Kraft lignin and lignosulfonates was conducted by Kopacic et al. [28], who found that such sizes improved the tensile index and contact angle, while reducing air permeation and water absorption. The use of a non-traditional lignin, i.e., the effluent from alkaline peroxide mechanical pulping, has also been demonstrated to improve the water resistance of corrugated paper [29]. Javed et al. further explored combinations of Kraft lignin with starch, glycerol, and ammonium zirconium carbonate, which were found to decrease water and oxygen permeation of the coated paperboard [30]. Internal sizing with lignin has also been studied in the context of added-lignin thermoformed pulps (ALTPs) [4]. Here, adding lignin significantly increased density while reducing water uptake. These effects were explained by the lignin melting under thermopressing conditions, filling in cavities and providing a tighter fit. The effect on contact angle was less pronounced, as the lignin was not concentrated on the surface as in previous surface sizing experiments. A subsequent study found that the reduced water uptake went hand in hand with reduced swelling of the lignocellulose fibers [12]. This indicated that fiber confinement was one of the governing mechanisms, hence illustrating lignin’s ability to act as a binder. Thermoforming with added lignin thus appears as an effective alternative to form three-dimensional products, which have the potential to replace single-use plastics. 

The concept of adding lignin to pulp-based materials has existed for some time; however, the combination of this concept with molded pulp is still quite new [4]. The use of high temperature and pressure can draw on the thermoplastic property of lignin, hence enabling it to flow into cavities and act as a binder. Recent investigations have added lignin to thermoformed pulp by internal sizing, coating, and impregnation [12]. Importantly, precipitating the lignin onto the fibers during pulping is a new concept, which has not been explored in this context. The purpose of this study was therefore to demonstrate the production of 100% renewable thermopressed specimens, which carries the potential to be applied in thermoformed pulp products to replace single-use plastics. To achieve this promising objective, a process was devised to obtain lignin-coated cellulose fibers and organosolv lignin. These components were used to make handsheets by wet-forming, and the properties of thermopressed materials were assessed. The potential synergistic effect of combining organosolv lignin and kraft pulp fibers was also assessed. The mechanical characteristics, surface morphology, chemical interactions, and water wetting properties of these materials were analyzed and discussed.

## 2. Experimental Section

### 2.1. Materials

Bleached softwood kraft pulp (NBSK) was provided by MoRe Research AB (Örnsköldsvik, Sweden). A detailed characterization of this pulp is given in a previous article [3]. The chemicals purchases were acetone (SA quality, >99.7%, ROMIL, Oslo, Norway) and sulfuric acid (AnalaR NORMAPUR, 98%, VWR, Oslo, Norway). Deionized water was used, if not specified otherwise.

### 2.2. Sample Preparation

Organosolv pulping was conducted by acetone pulping as described previously [31]. Here, a total of 100 g (dry matter weight) of Norwegian spruce chips were digested in a 50/50 (*w*/*w*) blend of acetone/water (7.5:1 liquid/wood ratio) and 1% sulfuric acid per dry wood weight. Impregnation of the chips was conducted at 6 bar for 10 min, after which the reactor was ramped up to 195 °C, holding the maximum temperature for 60 min. Afterwards, the cooking liquor was displaced with 700 mL of 20/80 (*w*/*w*) acetone/water, while maintaining the same temperature and pressure. This was followed by displacement and washing with water, going stepwise from the reaction temperature to 80 °C and finally to ambient temperature. The organosolv fibers were obtained from the residual solids. The organosolv lignin was precipitated from the cooled cooking liquor by addition of deionized water, which was conducted at a ratio of 3:1 water to liquor. The precipitated lignin was filtered by a glass microfiber filter (Whatman, cat. no. 1820 090, Sigma-Aldrich, Oslo, Norway). The filter cake was washed with 500 mL deionized water, extracted with an 80/20 (*v*/*v*) blend of acetone and water, dried first under ambient conditions and then in vacuo at 55 °C, and finally crushed to particles with a size of <297 µm (mesh 48 pass). The final yields were 56% organosolv fiber and 3% organosolv lignin per input material, where losses can be attributed to dissolved and dispersed matter in the displacement and washing liquid.

### 2.3. Handsheet Preparation

Prior to sheet formation, the organosolv or Kraft fibers were dispersed in hot water (>85 °C) according to ISO 5263-1:2004 [32], using a Lorentzen & Wettre disintegrator at 30,000 revolutions (Stockholm, Sweden). The pulp suspension was subsequently diluted to a consistency of 3 g/L. Handsheets were made in a custom-made sheet former. The measured width by height was 80 mm × 150 mm and a basis weight of 300 g/m^2^ pulp was targeted. The pulp fibers were either used directly, blended at a 50/50 *w*/*w* ratio, or sized with lignin. The sizing was conducted by adding 2.4 g organosolv lignin per sheet before sheet formation. An amount of 200 ppm cationic flocculant (PCB 20, Solenis Norway AS, Drammen, Norway) per dry pulp weight was added in addition, as this promoted adhesion of the lignin particles to the fibers and hence a more homogeneous distribution of lignin throughout the fiber network. After sheet formation, the handsheets were pressed between blotting papers at 2 MPa pressure and ambient temperature for 5 min. Afterwards, the handsheets were air-dried in a climate chamber (23 °C, 50% relative humidity) to about 90% dry matter content. Finally, each sheet was thermopressed between metal plates at 175 °C and 50 MPa for 10 min.

### 2.4. Analytical Procedures

Before each analysis of handsheets, these were acclimatized in a climate chamber in standard conditions (23 °C, 50% relative humidity) for at least 24 h prior to testing.

#### 2.4.1. Chemical Composition

The chemical composition of the raw materials was conducted according to the NREL method, i.e., by measuring acid-insoluble and -soluble lignin, as well as carbohydrate analysis [33]. Here, the material was first dissolved in concentrated sulfuric acid (72%) and incubated at 30 °C for 1 h. Afterwards, the dispersion was diluted to 4% and autoclaved at 121 °C for 1 h. The sample was then filtrated, where acid-insoluble lignin was measured gravimetrically and acid-soluble lignin was measured through UV/vis spectrophotometry. The carbohydrate composition was subsequently measured through liquid chromatography. The procedure for measuring acid-soluble lignin followed the TAPPI UM 250 standard [34], i.e., using the absorbance at 205 nm and an extinction coefficient of 110. The sugars in the filtrate were analyzed through HPLC. The procedure is also described in reference [31]. 

The cellulose $C_{cell}$ and galactoglucomannan $C_{galglu}$ (hemicellulose) content were estimated from the abundance of glucose $C_{gluc}$, mannose $C_{man}$, and galactose $C_{gal}$ according to Equations (1) and (2) [35].

(1) $$C_{cell}=C_{gluc}-1/3C_{man}$$

(2) $$C_{galglu}=4/3C_{man}+C_{gal}$$

#### 2.4.2. Fiber Size Distribution

The size distribution of fibers was tested in a Lorentzen & Wettre Fiber Tester Plus. For each run, 200 mL of a 0.05 wt.% fiber suspension was injected into the instrument and further diluted to 0.002 wt.%. Images were taken by a digital camera of the circulating sample, while the fiber geometries were analyzed by the instrument software. Fines were defined as fibers that are under 0.2 mm in length, while fibers were defined as having a shape factor of at least 50%. Objects measuring at least 75 µm × 75 µm were defined as wide objects and exempted from the results. The given percentages are hence solely with respect to the detected fibers and fines.

#### 2.4.3. Apparent Density

The handsheets were cut to clean corners and a defined area and were weighed, and the thickness was measured according to ISO 534 [36] at five predetermined positions. The area, thickness, and weight were then used to calculate the apparent density.

#### 2.4.4. Tensile Properties

Tensile testing was conducted on a Zwick material tester equipped with a 2.5 kN KAF-W force transducer (ZwickRoell, Ulm, Germany). Each handsheet was cut into stripes with a width of 15 mm, yielding 6–10 test stripes per sample. These stripes were then tested at an elongation speed of 100 mm/min.

#### 2.4.5. Scanning Electron Microscopy (SEM)

A Hitachi SU 3500 (Tokyo, Japan) low-vacuum microscope was used for taking SEM images. All imaging was performed in secondary electron imaging (SEI) mode after fixing the substrate in place and applying a layer of conductive gold. The acceleration voltage and working distance were 5 kV and 6–8 mm, respectively.

#### 2.4.6. Contact Angle

The contact angle was measured on a Dynamic Adsorption Tester (DAT) 112 from Fibro systems (Stockholm, Sweden). Long stripes of sample substrate were fed into the instrument, which applied 3–4 µL deionized water onto a new section of specimen. The droplet shape was then recorded using a high-speed camera, determining the contact angle from these images using the instrument software. Between 10 and 13 measurements were conducted per sample.

#### 2.4.7. Water Uptake

Five test pieces measuring 15 mm × 15 mm were cut out from each sample, equilibrated in standard conditions, and then immersed in deionized water for 24 h. Afterwards, the test pieces were wiped with dry laboratory tissue and weighed. The water uptake ($p_{water}$) was then calculated from the specimen weight before ($m_{50\%RH}$) and after immersion in water ($m_{water}$) as stated in Equation (3).

(3) $$p_{water}=\frac{{(m}_{water}-m_{50\%RH})}{m_{50\%RH}}$$

#### 2.4.8. Fourier-Transform Infrared Spectroscopy (FT-IR)

Attenuated total reflectance-Fourier-transform infrared spectroscopy (ATR-FTIR) was conducted on a PerkinElmer Spectrum 3 with Universal ATR Sampling Accessory (Shelton, CT, USA). The substrates were pressed onto the FTIR prism while performing 32 runs and averaged at a step rate of 4 cm^−1^. Correction for residual moisture was carried out using the instrument software. Each graph was baseline-corrected with a cubic spline and normalized via the C-H stretching band maximum in between 2800 and 3000 cm^−1^.

#### 2.4.9. Statistical Analysis

To compare the means of two individual measurements, the two-sided *t*-test was conducted according to Equations (4) and (5) given by Kalpic et al. [37]. Here, the variable T was calculated from the sample means ${\bar{X}}_{i}$ with standard deviation $S_{i}$ and sample size $n_{i}$. By combining the degree of freedom ν with the t-distribution as published in relevant textbooks [38], the null rejection or approval of the null hypothesis with confidence interval α can be tested.

(4) $$T=\frac{{\bar{X}}_{1}-{\bar{X}}_{2}}{\sqrt{\frac{S_{1}}{n_{1}}+\frac{S_{2}}{n_{2}}}}$$

(5) $$\nu =\frac{{\left(g_{1}+g_{2}\right)}^{2}}{\frac{g_{1}^{2}}{n_{1}-1}+\frac{g_{2}^{2}}{n_{2}-1}};\;\;\;\;\;g_{i}=\frac{S_{i}^{2}}{n_{i}}$$

## 3. Results and Discussion

### 3.1. Raw Materials

The compositional changes during organosolv pulping are discussed first, as this highlights the effect of the fractionation procedure on the resulting product. The sugar distribution and biopolymer contents are listed in Table 1. Here, the spruce wood contained around 40 wt.% cellulose, 18 wt.% hemicellulose, and 32 wt.% lignin, which are typical values found for softwood [39]. After organosolv pulping, the hemicellulose content was greatly reduced (1.6 wt.%), whereas the relative cellulose content increased to 67.5 wt.%. The lignin content decreased only by about 4.5 wt.%, which suggests that lignin removal was likely limited and that lignin reprecipitated onto the fibers during solvent displacement. The lignin-to-cellulose ratio was 0.8 before and 0.4 after pulping. This further illustrates that the organosolv pulping was effective at liberating cellulose and removing most of the hemicelluloses. The extractives and ash content of the spruce wood chips were determined as 1.0 wt.% and 0.2 wt.%, respectively.

**Figure 1:**
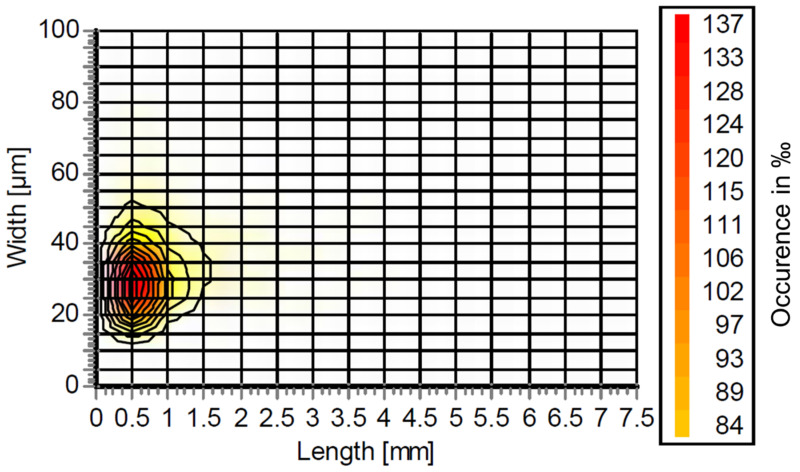
Fiber size distribution of organosolv fibers.

The results from the Fiber Tester are plotted in Figure 1. As can be seen, there are predominantly short fibers and fines with a length between 0 and 1.5 mm and a width between 10 and 50 µm. Here, the greatest abundance is at a length and width of 0.5 mm and 30 µm, respectively. Compared with previous results in CTMP or Kraft pulp [3,4], the long fiber fraction at >1.5 mm length is lacking. This is also mirrored by the data in Table 2 which compares the Kraft and organosolv fibers. Here, the mean fiber width is almost the same, whereas the mean fiber length is approximately four times larger for the Kraft pulp. Along with this smaller fiber size, the fines content of organosolv fibers is significantly higher at 82.3% as compared to 15.4% for Kraft. Compared with our previous publication, the dimensions of the organosolv fibers are closer to those of fibers with hydrolysis lignin, which were mechanically treated in a blender [4].

**Figure 2:**
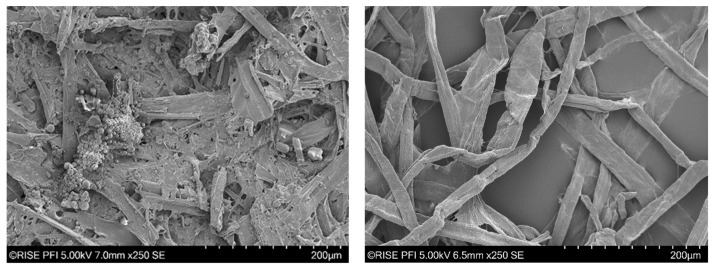
SEM images of organosolv (**left**) and Kraft fibers (**right**).

SEM images of individual fibers at 250× magnification are shown in Figure 2. As can be seen, the Kraft pulp exhibits long fibers with straight or kinked shapes. Compared with the organosolv fibers, the Kraft fibers show only few objects below 100 µm and a smooth fiber surface. The organosolv fibers in comparison exhibit shorter fibers and more inhomogeneous fiber surfaces. In particular, the presence of sphere-shaped objects with varying size on the surfaces of the fibers can be noted. These spheres have been identified as lignin particles, which stem from reprecipitation from the cooking liquor [40]. The SEM images therefore support the conclusion drawn from the compositional changes in Table 1, i.e., reprecipitation of lignin occurring during solvent displacement and washing of the fiber fraction. Moreover, the qualitative images are in agreement with the observations made by the Fiber Tester device.

### 3.2. Surface Properties of the Thermopressed Handsheets

Two approaches were used to characterize the surface properties of the thermopressed handsheets, which are mapping the surface morphology using SEM and the surface chemistry using ATR-FTIR.

**Figure 3:**
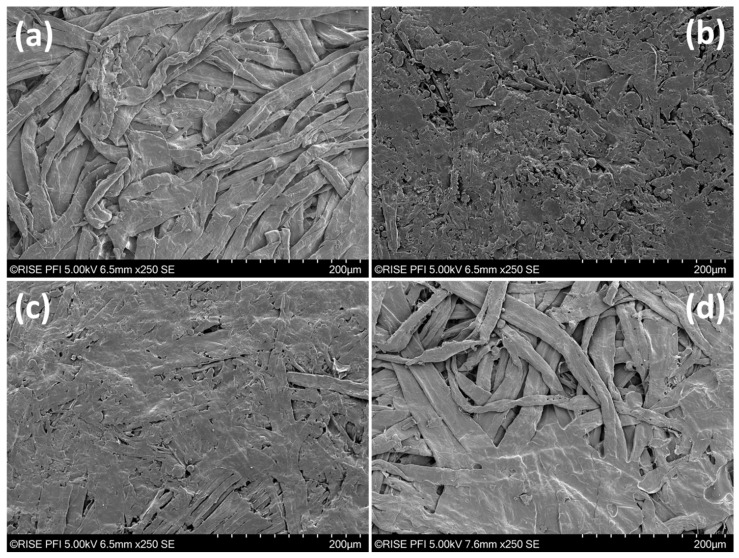
SEM images of thermopressed handsheets. (**a**) Kraft fibers, (**b**) organosolv fibers, (**c**) Kraft + organosolv fibers, and (**d**) Kraft fibers + organosolv lignin.

The results of SEM imaging are shown in Figure 3. Here, the substrate with only Kraft fibers exhibited similar shapes as in Figure 2. The main differences are more collapsed fibers that are tightly packed. The organosolv fibers, on the other hand, showed a dense and relatively smooth surface after thermopressing. Individual fibers are difficult to recognize and there are significantly fewer cavities visible at the surface than for the Kraft fibers. Such morphologies could be due to the smaller fibers, but also the presence of lignin on the fiber surfaces. The blend of Kraft and organosolv fibers mirrored to a large extent the surface of organosolv fibers only. A few Kraft fibers are visible within the organosolv fiber surface; however, the packing at the surface appears far denser than for the Kraft fibers alone. Finally, adding organosolv lignin to the Kraft fibers provided areas and spots with very smooth surfaces. Figure 3d displays an image taken of an area, which comprised both visible Kraft fibers and organosolv lignin. Both areas with only Kraft fibers at the surface but also smooth surfaces were observed. The thermopressing temperature was high enough to melt the lignin, hence allowing it to flow into cavities. The latter was the predominant configuration, indicating that most of the surface was partially covered with organosolv lignin. It appears that the sizing with organosolv lignin was thorough, but also inhomogeneous to a certain extent. The application of the lignin as particles with a diameter of <297 µm (mesh 48 pass) likely contributed to such a spot-wise occurrence. A more homogeneous distribution could be achieved by applying colloidal lignin, yet this can cause plugging during drainage and web formation, hence lowering the achievable tensile strength [12].

**Figure 4:**
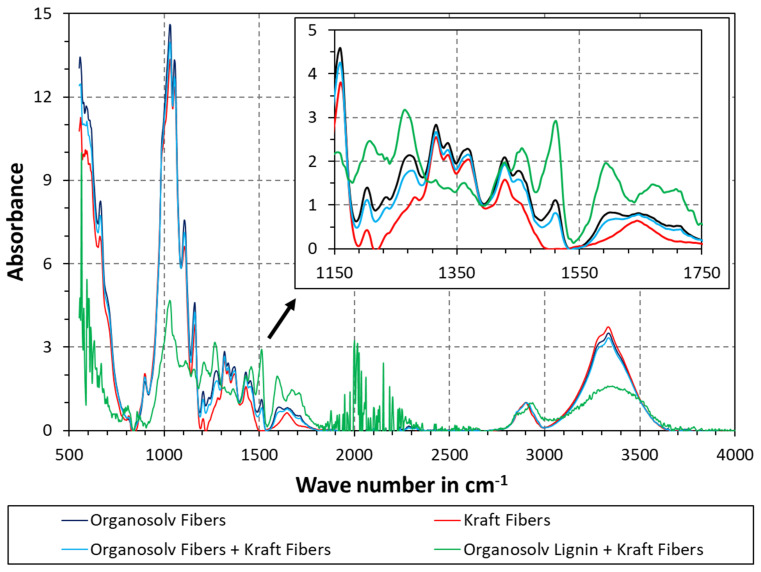
ATR-FTIR of thermopressed fiber materials. Each graph was baseline-corrected by a linear spline and normalized via the maximum of the C-H stretching band between 2800 and 3000 cm^−1^.

ATR-FTIR was additionally used to probe the surface chemistry. To facilitate better comparison, the data in Figure 4 were baseline-corrected and normalized as described in the Methods section. The peak between 3100 and 3600 cm^−1^ corresponds to the O-H stretching band. As can be seen, the highest O-H was found for Kraft fibers, whereas the lowest O-H was seen for Kraft fibers with added organosolv lignin. Cellulose is naturally rich in O-H groups, while the content of these groups for lignin is lower. The Kraft fibers were virtually free of lignin, so the observed trend makes sense. In addition, some lignin was visible on the surface of the organosolv fibers (see Figure 1). Accordingly, samples with organosolv fibers also exhibited lower O-H than the pure Kraft fibers in Figure 4. The presence of lignin at the fiber surface is also highlighted by the aromatic stretching band at 1505–1510 cm^−1^. Kraft fibers with organosolv lignin exhibited the highest peak here, followed by organosolv fibers. The difference in peak height between organosolv fibers alone and Kraft fibers with organosolv fibers is smaller than the difference with Kraft fibers alone. The peak right above 1700 cm^−1^ should also be mentioned, as this corresponds to the C=O stretching of unconjugated ketones, carbonyls, and in esters. This peak was pronounced for the sample containing organosolv lignin, and visible for the samples containing organosolv fibers, but not for the Kraft fibers. Moreover, the region between 1400 and 1450 cm^−1^ is almost identical for all samples. The C-H deformations are located here, so the overlapping signals make sense as normalization was conducted at the C-H band at a higher wave number. Overall, the spectra of organosolv fibers with and without Kraft fibers are very similar, which supports the observation from Figure 3c, i.e., that there are predominantly organosolv fibers at the surface of the blend. The spectrum of Kraft fibers with added lignin is qualitatively different from all other spectra, highlighting a significant difference in surface chemistry. Yet, the spectra of samples with organosolv fibers appear in the middle of Kraft fibers with or without added lignin. Again, this supports the interpretation of reprecipitated lignin on the surface of organosolv fibers. Moreover, the pulping with organic solvents may also yield compositional differences, for example by removing less hemicelluloses than during alkali pulping. Interactions of the pulping solvent with the pulp could also occur; however, the data at hand are insufficient to reach such a conclusion.

### 3.3. Density and Tensile Properties

**Figure 5:**
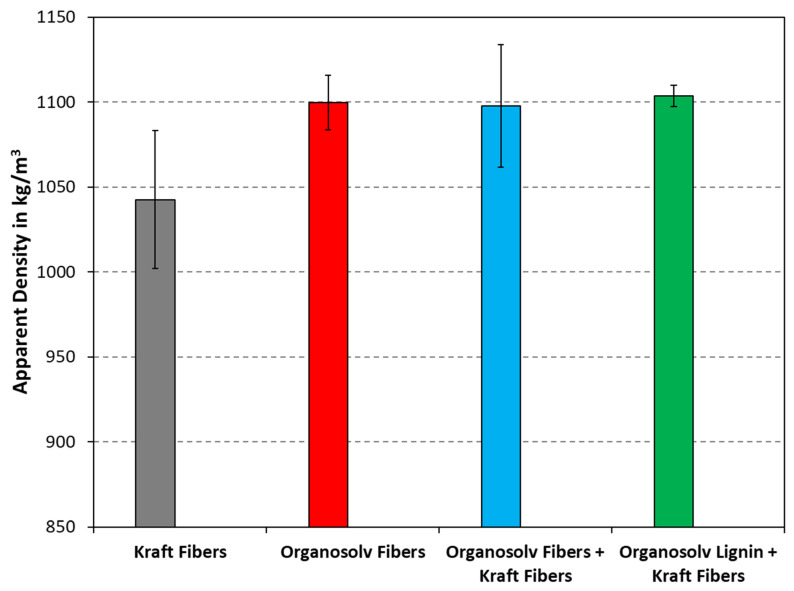
Densities of thermopressed fiber materials with different compositions. The columns and error bars indicate the average and standard deviation, respectively.

The densities of thermopressed fiber materials are plotted in Figure 5. As can be seen, the density of pure Kraft fibers appeared lower, whereas the products from organosolv pulping and their mixtures with Kraft pulp were similar. The latter contained technical lignin, be it from reprecipitation or addition, which can reportedly fill in cavities, thereby promoting a higher density [4]. Still, when performing the two-sided *t*-test with a confidence level of 95%, the zero hypothesis was rejected, i.e., the difference between the densities was not statistically significant.

**Figure 6:**
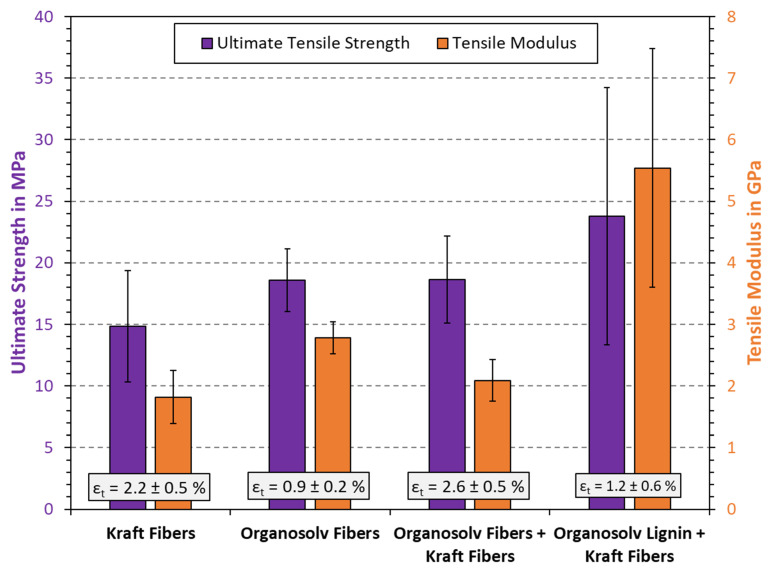
Results from tensile testing of thermoformed fiber materials. The columns and error bars indicate the average and standard deviation, respectively.

The outcome of tensile testing is displayed in Figure 6. Interestingly, the Kraft fibers alone yielded the lowest tensile strength of all tested samples. Kraft pulp is commonly used as reinforcement in applications such as cardboard and packaging. However, in this study, the pulp was not beaten before use. The beating of kraft pulp fibers affects the mechanical properties of handsheets [41]. Stiff fibers and the absence of secondary fines may therefore account for relatively poor bonding and hence a lower tensile strength, as compared to what could be achieved [42].

The organosolv fibers yielded at stiffer material with lower extensibility, but also greater tensile strength than the Kraft fibers. Secondary fines can contribute to improved strength [43]; however, it remains uncertain if the fines in the organosolv fibers are primary or secondary fines. At the same time, the lack of long fibers will reduce extensibility, as there is little room for the matrix to stretch. Furthermore, the addition of lignin has been shown to increase stiffness and reduce the elongation at break (ε_t_) for thermoformed pulp materials [4]. Combining Kraft and organosolv fibers increased both the tensile strength and elongation at break, if compared to Kraft pulp alone. This suggests that combining long and short lignin-coated fibers could provide better bonding, while still allowing the network to deform to a certain extent. Interestingly, the stiffness (tensile modulus) was in between that of the two individual fibers. The better tensile strength of materials including organosolv fibers is likely also related to the density, as density can correlate linearly with tensile strength for chemical pulp [3]. Finally, the addition of organosolv lignin considerably improved the mechanical properties of the Kraft pulp. An increase in tensile strength from 15 MPa to 24 MPa and an increase in modulus from 1.8 GPa to 5.5 GPa were observed. As recently reported, such a change can stem from the lignin filling in cavities, providing a tighter fit and a greater area of bound material [4,12]. A statistical analysis was also conducted, which used the two-sided *t*-test. Here, it was found that the difference of Kraft fibers to all other substrates and blends was statistically significant at a confidence interval of α=90%. Relating the other values to each other was not found to be statistically significant, even at α=90%.

Importantly, the improvements noted in Figure 6 are so far unprecedented for added-lignin thermoformed pulps (ALTPs). Mechanical fibers were employed in previous studies [4,12], suggesting that softwood Kraft pulp may be even more suited for ALTP. Both surface chemistry and the fiber dimensions may play a role in this, which can further affect fiber–fiber adhesion and binding. Stiffness increases are, however, more evident after adding lignin, having been shown to add stiffness to composite materials in general [44,45]. One limitation of adding organosolv lignin to Kraft fibers was a reduction in the elongation at break. This behavior was expected, considering the brittle nature of lignin. All in all, the results illustrate that the products from organosolv pulping, i.e., both the obtained fiber and lignin fractions, have beneficial effects when added to thermopressed handsheet specimens. A higher material strength and stiffness are further useful in material applications, in particular packaging, as this will allow a smaller material width and hence lower overall weight.

### 3.4. Water Interactions

**Figure 7:**
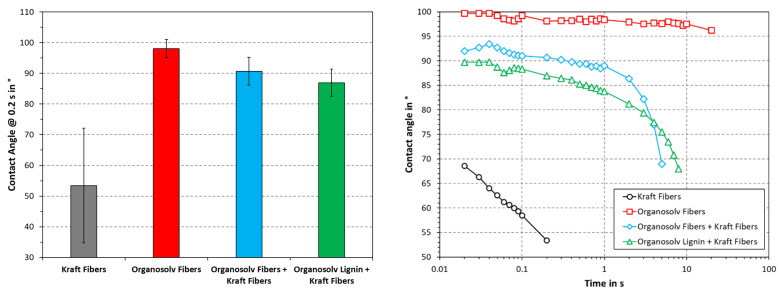
Aqueous contact angle with respect to time (**right**) or at 0.2 s. The columns and error bars indicate the average and standard deviation, respectively.

One of the challenges of cellulose-fiber-based materials is that these are prone to wetting. Water absorption can induce fiber swelling and interfere with inter-fiber adhesion, e.g., by decoupling hydrogen bonds. Paper and pulp products are hence deteriorated by the presence of water, as the cohesion within the fiber network is largely lost. Thermopressing can reportedly render such materials less hydrophilic, reducing fiber swelling and wetting [12]. The aqueous contact angle was therefore studied, as this provides a measure for the surface hydrophobicity and one-dimensional wetting. As can be seen in Figure 7, the lowest contact angle was obtained by Kraft fibers alone. Organosolv fibers, on the other hand, exhibited the highest and most stable contact angle. A combination of dense surface and residual lignin on the fibers (see Figure 2 and Figure 3) could likely be an explanation for this. The addition of organosolv fibers or organosolv lignin to Kraft fibers increased the contact angle, as compared to Kraft fibers alone. Initially, the contact angle of Kraft fibers with organosolv fibers is greater, but at ≥4 s, the Kraft fibers with organosolv lignin show higher values. The hydrophilicity of bleached softwood Kraft likely counteracts the reduction in wetting of the organosolv products. Still, the organosolv fibers behaved like a hydrophobic material in this test (contact angle > 90°), which suggests a high potential for applications in moist conditions. Compared with mechanical pulp thermopressed in the same conditions (175 °C, 75 MPa), the contact angle of the Kraft fibers was significantly lower [12]. This difference is likely due to a difference in chemical composition, as the Kraft fibers are virtually lignin-free, whereas the mechanical pulp contained near-native amounts of lignin [3]. During thermopressing, high temperatures can yield a softening of the lignin, making it moldable. The absence of natural lignin hence appears to greatly reduce the ability of thermopressed substrates to withstand wetting.

**Figure 8:**
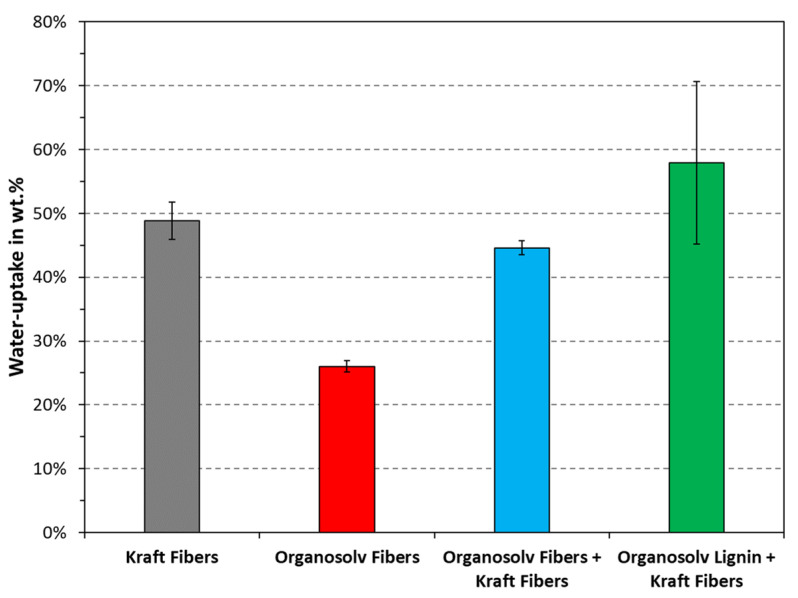
Water uptake of the tested materials. The columns and error bars indicate the average and standard deviation, respectively.

The water uptake quantifies the ability of a substrate to absorb water and swell, as opposed to the contact angle, which probes the surface hydrophobicity and one-dimensional mass transport. The measured water uptakes are plotted in Figure 8. Interestingly, this test showed that the water uptake for Kraft fibers with and without organosolv lignin was similar. Added lignin actually resulted in a higher value; however, no statistically significant difference was found when performing the two-sided t-test, even with a confidence level of α=90%. This would suggest that the lower hydrophilicity, as noted in Figure 7, was mainly due to the organosolv lignin providing a mass-transfer barrier. All other differences were found to be statistically significant at α=96%, except for comparing organosolv fibers + Kraft fibers with organosolv lignin + Kraft fibers. The lowest water uptake was noted for the organosolv fibers, followed by Kraft fibers with organosolv fibers. It appears that the materials formed by organosolv fibers are the least hydrophilic as documented by both contact angle and water uptake. It remains open whether this property arises from the inherent composition and properties of the fibers, or if this fiber type is particularly well suited for thermopressing. 

It can be concluded that organosolv fibers appeared particularly suited for potential thermoformed products that require resistance to wetting, e.g., trays for food packaging. Materials for food packaging usually include, e.g., fossil low- and high-density polyethylene (LDPE and HDPE). The strength and modulus of LDPE and HDPE are roughly between 10 and 30 MPa and 0.3 and 1.0 GPa, respectively [5], which are comparable to the mechanical properties obtained in this study (Figure 6). Although the elongation of the thermopressed handsheets are considerably lower (<10%) than those of PE materials (>500%), this is not considered a major drawback, as a high stiffness is particularly considered crucial food packaging and trays [46]. Hence, these results may contribute to developing materials from lignocellulose fibers, which are more suited for moist applications than traditional paper and pulp products.

## 4. Conclusions

In this study, new materials were studied that combined organosolv pulping with thermopressing. Both the fiber fraction and the lignin obtained by organosolv pulping were attempted in combination with softwood Kraft pulp. After characterization of the input fibers, laboratory handsheets were formed and thermopressed at 175 °C and 50 MPa. The surface morphology and chemistry were studied using SEM and ATR-FTIR, respectively, after which tensile testing, contact angle, and water uptake measurements followed.

Organosolv pulping produced a fraction with shorter fiber length and a significantly higher fines content than the Kraft pulp. The fiber surface was furthermore irregular and marked by reprecipitated lignin particles. After thermopressing, the organosolv fibers yielded a smoother surface than the Kraft fibers with visibly smaller pores. Combinations of organosolv and Kraft fibers mirrored the properties of both pulps. The tensile strength and stiffness of organosolv fibers were better than those of the Kraft pulp, albeit at a lower elongation at break. Blending both fibers combined the higher stiffness and strength with greater extensibility, yet the best results were obtained for Kraft fibers with added organosolv lignin. It appears that the added lignin improved fiber bonding, while better cohesion due to a greater bound area could be an additional mechanism. The greatest potential for organosolv fibers was, however, identified as hydrophobization or reduced wetting. Thermopressed organosolv fibers showed contact angles stable at >95°, which likely stemmed from the lignin and the dense surface. The water uptake was also lowest for the organosolv fibers at only 26%, as compared to approximately 50% for the Kraft fibers. The addition of organosolv fibers or lignin to Kraft fibers increased the contact angle; still, the effect on water uptake was less pronounced.

In conclusion, great potential was found for the fiber fraction from acetone pulping. Reprecipitation of the lignin particles on organosolv fibers appeared to improve the properties of thermopressed handsheets, with potential to be used in thermoformed pulp products. Not only were the stiffness and strength better than for the reference fibers, i.e., softwood Kraft pulp, but the susceptibility to wetting was greatly reduced. This entails a promising potential for the product streams from organosolv pulping, which could contribute to develop more sustainable materials and packaging solutions.

## Figures and Tables

**Table 1:** Raw material composition.

	Spruce Wood Chips	Organosolv Fibers
Glucose (wt.%)	43.79	67.89
Xylose (wt.%)	4.59	2.09
Arabinose (wt.%)	1.28	0
Galactose (wt.%)	1.40	0
Mannose (wt.%)	12.48	1.18
Cellulose (wt.%)	39.6	67.5
Hemicellulose (galactoglucomannan) (wt.%)	18.0	1.6
Lignin (acid insoluble + acid soluble) (wt.%)	31.5	27.0

**Table 2:** Average fiber length, width, and fines content of the Kraft and organosolv fibers.

		Kraft Fibers	Organosolv Fibers
Mean fiber length	mm	2.018 ± 0.042	0.565 ± 0.017
Mean fiber width	µm	27.6 ± 0.2	29.5 ± 0.2
Mean fines content	%	15.4 ± 0.8	82.3 ± 0.2

## Data Availability

Data can be made available upon request.
